# Designing a guideline for empowering married adolescent girls in reproductive health: a mixed-method study protocol

**DOI:** 10.1186/s12978-018-0654-9

**Published:** 2018-12-17

**Authors:** Shirin Allahverdizadeh, Shahnaz Kohan, Ziba Farajzadegan, Morteza Ghojazadeh

**Affiliations:** 1 Student Research Center, Faculty of Nursing and Midwifery, Isfahan University of Medical Sciences, Isfahan, Iran; 2 Nursing and Midwifery Care Research Center, Faculty of Nursing and Midwifery, Isfahan University of Medical Sciences, Isfahan, Iran; 3 Community and Preventive Medicine Department, Medicine Faculty, Medical Sciences University Of Isfahan, Isfahan, Iran; 4 Research Center for Evidence- Based Medicine, Health managment and Safety Promotion Research Institute, Tabriz University of Medical Sciences, Tabriz, Iran

**Keywords:** Empowerment, Guideline, Need, Married adolescent girls, Reproductive health, Mixed- method study

## Abstract

**Background:**

Investing in adolescent’s health, especially, the role of girls in community health and future generations is one of the most important strategies of the Millennium Development Goals. In this regard, supplying adolescents’ special needs including access to educational, health and counseling services for promoting reproductive health have been emphasized. About 36% of registered marriages in Iran are under the age of 19 though, reproductive health services based on married adolescent girls` needs in social-cultural context were not predicted in national health system. Therefore, this study aim was designing a guideline for empowering married adolescents in reproductive health.

**Methods:**

This is a sequential exploratory Mixed-method study conducted in three consecutive phases. The first phase, with a qualitative approach, explores needs, barriers and strategies for empowering married adolescent girls in reproductive health. In the second phase, a systematic review will be conducted to identify the recommendation and strategies for empowering married adolescent girls in reproductive health in other countries. Finally, in third phase, data from qualitative study and systematic review are emerged and the most important solutions and recommendations related to the issue are extracted and the final guideline is adapted by the experts.

**Discussion:**

This study is attempting to provide a guideline containing comprehensive recommendations for health system` policy makers and providers in order to empowering adolescent girls in reproductive health.

## Plain English summary

Investing in adolescent’s health, especially, the role of girls in community health and future generations, is the most important. In this regard, supplying adolescents’ special needs including access to educational, health and counseling services for promoting reproductive health have been emphasized. About 36% of registered marriages are under the age of 19 in Iran, though, reproductive health services based on married adolescent girls` needs in social-cultural context were not predicted in national health system. Therefore, the results of this study offer a guideline for empowering married adolescents in reproductive health. This is a sequential exploratory Mixed-method study conducted in three consecutive phases. The first phase, with a qualitative approach, explores needs, barriers and strategies for empowering married adolescent girls in reproductive health. In the second phase, a systematic review will be conducted to identify the recommendation and strategies for empowering married adolescent girls in reproductive health in other countries. Finally, in the third phase, data from qualitative study and systematic review are emerged and the most important solutions and recommendations related to the issue are extracted and the final guideline is adapted by the experts. Therefore it is expected conducting a mixed method study to provide a guideline containing comprehensive recommendations for health system` policy makers and providers in order to empowering adolescent girls in reproductive health.

## Background

Around one-fifth of the world population has between 10 and 19 years old and 85% of them are living in developing countries. In the world, about 16 million girls aged 15–19 get pregnant including 11% of all births and 95% of these births occur in low and middle income countries [1–3]. Early marriage in the Iranian society is deep-rooted. In 2011, Iran statistics showed that about 36% of registered marriages were girls aged fewer than 19, which increased in 2013. With rising young girls` marriage, the possibility of having children will increase. As a result, the five-year statistics (2009–2013) in Iran showed that mothers under the age of 19 make up about 8–9% of all births. However, reproductive health services based on married adolescent girls` needs in social-cultural context were not predicted in national health system [4].

For most of these adolescents, pregnancy is both unwanted and unplanned. Unwanted pregnancy may be under pressure due to early marriage and forced childbearing, while most of them do not know how to prevent pregnancy or have not access to contraception methods [5–8]. Adolescents also do not have the ability to refuse forced sexual relations [9, 10]. Therefore, for millions of teenagers, these early unwanted pregnancies are accompanied with fear and pain, and between 2 and 4 million adolescents annually use unsafe abortions representing 14% of the total abortions, and its complications are more pronounced in adolescents. Therefore, prevention of unwanted pregnancy in adolescents is a priority [11].

Reproductive health is related to physical, emotional and social well-being of the reproductive and sexual health emphasizes a positive and respectful approach to sex and an enjoyable and safe experience, without compulsion, discrimination, and violence. Consequently, attention to all aspects of sexual and reproductive health, especially adolescent reproductive rights, was addressed in the ICPD Statement.

Furthermore, empowerment of girls as a prerequisite for promoting reproductive health was proposed in international agreements, including ICPD, MDG and sustainable development goals (SDG), to promote gender equality, empowerment of girls and eliminate all forms of violence for equal access to resources and ensure their basic right to make decisions about their lives [12].

Investing in the reproductive health of this age group is one of the main paths for achieving the Millennium Development Goals as their role in community health and next generations [13]. Therefore, provision of adolescent reproductive health needs is becoming more important and it is necessary to identify barriers to girls’ reproductive health [9].

To empower adolescents in reproductive health, their needs must be identified on the basis of cultural and social context and sensitive services should be provided in accordance with the biological, cognitive and psychosocial structures. They require appropriative reproductive health services to meet their needs, which should be provided in the framework of adolescent-friendly services. In some countries, there are programs and interventions on the reproductive health services for adolescents. Most of these programs are not based on reproductive needs of adolescents. There is also little information in Iran about the reproductive health of adolescent married girls. Therefore, this study aims to developing a guideline for empowering married adolescents girls in reproductive health tailored to the cultural context of their community and the specific needs of this vulnerable group.

## Objective

### The first phase: Qualitative study

1. Exploring the participants` experiences about reproductive health needs of married adolescent girls.

2. Identifying barriers to married adolescent girls` access to reproductive health services.

3. Exploring strategies to empowering married adolescent girls in reproductive health.

### The second phase: Systematic review

1. Determining the reproductive health needs of married adolescent girls.

2. Extraction strategies and recommendation to empowering married adolescent girls in reproductive health.

### The third phase: Designing the guideline

1. Designing a preliminary guideline for empowering married adolescent girls in reproductive health.

2. Adaptation guideline by using RAND/ UCLA Appropriateness Method technique.

## Methods/design

This is a sequential exploratory study which approved by Isfahan University of Medical Sciences Ethics Committee (IR.MUI.REC.1395.3.897). In the first phase, using a qualitative approach, the needs, barriers and proposed strategies for empowering married adolescence girls in reproductive health will be explored. In the second phase, using a systematic review, the needs and recommendation for empowering of adolescent girls in reproductive health in other countries will be identified. In the third phase, by emerging the result of the previous studies, the most important needs, recommendation and strategies related to the reproductive health of the married adolescents will be identified and prioritized. Then, a preliminary guideline will be designed based on these data. Then, the opinion of experts about this guideline will be collected by using RAND/ UCLA Appropriateness Method technique.

### Phase I: Qualitative study

The first phase of this study is designed to answer the question “What are the needs, barriers and ways to empower married adolescent girls in reproductive health?” In this research, qualitative study will be carried out using qualitative content analysis method [14].

### The participants in the first phase (qualitative)

The participants of this study include Iranian married adolescent girls, their families (Adolescent parents, spouses and parent in law), policymakers and reproductive health professionals with at least 2 years experiences in providing marriage counseling, family planning, prenatal and childbirth care for adolescences who volunteer to participate in this study.

### Research environment

Selection of participants is conducted in their working environment (ministries of health, deputy of health and medical centers, health centers, marriage counseling centers, schools, universities, etc.) or in places preferred by the participants.

### Data collection in the first phase (qualitative)

With purposive sampling and maximum variation, eligible participants invite to join the research. Then, researcher introduce goals and method of research, after obtaining written informed consent from the participants, will be interviews at the time and place most convenient for the participant. Also they were given the option to withdraw from the study at any time. The data will be collected through in-depth, semi-structured, open-ended, and peer-reviewed interviews until data saturation. After completing each interview session, all interviews will immediately be transcribed. Before transcribing the audio file, the interviewer will listen carefully to the interviews several times. After detailed study and initial analysis of the text of each interview, the next interview will be scheduled. Data collection will continue as long as the data saturation is achieved, which means that no new data is extracted.

### Data analysis of the first phase (qualitative)

In the present study, qualitative conventional content analysis method will be used. The researcher will listen to the interview immediately after recording each individual interview, after finding a general overview of the interviews, the interviews will be transcribed word-by-word. Then, the sentences and the important phrases will be underlined, and the main ideas derived from them, labeled as codes. Then, similar codes will be merged and the initial classification will be performed. The process of data reduction will continue in all the analysis units in order to form categories. In this way, the data will be categorized as general, conceptual and abstract. Several strategies will be used for insuring the rigor of data, Participants will be selected with maximum variation; sufficient time will be assigned for data collection and integrated several data collection methods (interviewing and field notes). Some of the extracted data and codes will be reviewed by the participants and research partner. Finally, results of the study will be checked by some individuals (same the participants) who did not participate in the study, for assessing agreements between their experiences and the results of the research.

### Trustworthiness

To ensure the accuracy of the study and the reliability of the results, four of the proposed benchmarks of Polit &Beck: credibility, dependability, transferability and confirmability, will be used. In the present study, in order to increase the validity of the study, participants will be selected with maximum variation, sufficient time will be assigned for data collection,multi session deep interviews will be conducted at different times and locations, and the integration of multiple data collection methods, such as interviewing and taking notes in the field will be used. The method of reviewing by the participants will be used to verify the accuracy of the extracted data and codes or to modify them. In order to validate the findings, the external method’s observer will be used to investigate their probable [15].

### Phase II: Systematic review

In this phase, the needs, recommendation and strategies for empowering married adolescent in reproductive health will be extracted from the literatures by narrative review. The process of searching, analyzing data, and reporting the results in this study is based on the Prisma Standard Guide [16].

### Search strategy

In order to search all aspects of the topic, first, the “PICOT” will be determined as “what recommendations have been made so far to empower married adolescents girls in reproductive health”?

P= Married adolescent girls

I= Recommendation

C= National maternal care and reproductive health guideline

O= Empowerment in reproductive health

T=2000- 2018

In this phase of the research, resources and electronic databases that are available will be used to collect the required data, including: Cochrane, Pubmed, Embase, Scopous, and Google scholar, Ovid, Web of science, Science direct, SID, IranDoc, Magiran and IranMedex. For Gray Lectures, ProQuest, sites for endnotes, conferences, and abstracts will be used. The selected timeframe will be January 2000 to 2018. Articles will be searched using words matching Mesh. Key words and synonyms will be identified separately based on the clinical question. Selected keywords will be categorized into two groups:

- Terms which described the group of persons involved: Female Married Adolescent OR female Married teenagers, Adolescent pregnancy OR teen pregnancy OR teenage pregnancy, Adolescent mother OR teenager mother OR young mother

b) Terms that described type of services used: Reproductive health service, Maternal health, antenatal care, prenatal care, postnatal care, Skilled birth attendant, delivery, obstetric care, neonatal care, attachment

Using AND operators, the keys words will be combined as (individuals) and (service) in the group, and the search strategy will be compiled and run on specialist search bases (Table 1).

**[Table 1]:** Core Database Search Strategy

Focus	Search terms
(1) Population: adolescents AND	“Adolescent” OR “married adolescent” OR “teen” OR “married teen” OR “young women” OR “Adolescent pregnancy” OR “teen pregnancy” OR “teenage pregnancy” OR “Adolescent mother” OR “teenager mother” OR “young mother”
(2) Health services	
	“adolescent health services” OR “reproductive health services” OR “Family planning services” OR “Maternal health care” OR “antenatal care” OR “prenatal care” OR “postnatal care” OR “Skilled birth attendant” OR “delivery” OR “obstetric care” OR “neonatal care”.

All descriptive, interventional studies, systematic review in English or Persian language will be evaluated based on the needs, recommendation and strategies for empowering married adolescent girls in reproductive health. These articles will be entered in the study from 2000 until 2018 without limitation. In the first stage, title screening and in the second stage, abstract screening will be conducted by two experienced researchers. Studies that are unrelated or have inadequate data are out of time, and repetitive cases will be excluded from the study. If there is only an abstract of the articles available, the corresponding author will be contacted by e-mail three times for full text. If the access to full text is not possible, the article will be deleted. To avoid risk of bias within the studies, the study will be conducted by two researchers independently.

### Quality assessment

Critical Appraisal Skill Programs (CASP) checklist will be used for quality assessment of selected studies [17]. Then the cumulative quality scores of each study converted to percentage quality scores. Papers classified into high quality, when scored ≥70%, medium quality, if the scored from 50 to 70% and low quality if the scored < 50%.

### Data extraction

The data of the selected articles included in the study will be individually extracted by two researchers and are grouped in a form of table, which includes: the name of corresponding author, the year of the article publication, the method of search, the needs, recommendation and strategies for empowering married girls in reproductive health. It should be noted that if there is a difference in the extraction of data, agreement will be reached with the discussion**.**

### Data synthesis

For the synthesis of results, the following approach will be used. The thematic synthesis method allows us to find all similarities and differences of the various studies entered in the research [18]. To achieve this, according to the results of the present study, 2 themes will be predicted:

1. The reproductive health needs of married adolescent girls.
2. Recommendation and strategies to empowering married adolescent girls in reproductive health

### Phase III: Designing a guideline for empowering married adolescent girls in reproductive health

In this stage of the research, the needs will be extracted from qualitative study and systematic review, and will be put in matrix for prioritization by experts (obstetricians, reproductive and sexual health PhD, maternal health policy maker, Youth Health policy maker) which rating of each needs based on the four criteria including cost, time spent, and the place of execution and the feasibility. After prioritization, recommendation for each needs put in an early draft guideline and experts make decision by rating from 1 to 9. Based on these criteria, score 1 means that the expected disadvantages are greater than its advantages, and 9 means that the expected advantages are far greater than its disadvantages. Then, guideline will be sent to experts who desire to participate study, as panel members. To reach a consensus among experts RAND/ UCLA Appropriateness Method technique, will be used [19].

### RAND/ UCLA appropriateness method technique

In the first consensus round, the ranking of recommendation or services in the guideline will be carried out by experts individually without any interaction. For each recommendation, members of the panel will rate on a scale of 1–9. Recommendations that receive mean scores of 7 to 9 agreed by at least 70% of the experts will be considered as appropriate recommendations. Recommendations that receive mean scores of 1–3 agreed by less than 50% of experts will be considered inappropriate. Recommendations with a score of 4–6 agreed by more than 50% and less than 70% of the experts will be considered as recommendations that are uncertain. The researcher then examines the scores and calculates the mean scores for each service separately, and will prepare a new questionnaire, which will include the mean scores given by the team of experts, for each recommendation, in order to present.

In the second round, panel members will come together with research team in a meeting. Experts discuss the scoring of the each recommendation especially about uncertain scores. The focus will be on areas of conflict and there is an opportunity to changing the principal list of recommendations or services and final decision will be made. After applying all the corrections, the final guideline to empowering married adolescents in reproductive health in context of Iran health system will be developed.

## Discussion

Early marriage for girls is a barrier to educational, economic and social opportunities, they have low power compared to adult girls and often cannot decide on their own [20, 21].

They usually get pregnant immediately after marriage under the pressure of family and community. This is an unwanted pregnancy for most adolescents. Various studies have shown that the risks and complications of pregnancy in adolescents are very high. While most of them do not have enough information on how to prevent pregnancy, they are unable to refuse forced sex, and their reproductive health is endangered due to the gap between health knowledge and the performance of adolescents after the beginning of marital life [22, 23]. While international agreements emphasize promotion of reproductive health and well-being of adolescent girls and their role in the health of community and next generations [2, 12], meeting and satisfying their reproductive health needs in society is ignored [24, 25].

Regarding the status of girls in the community and the importance of their reproductive health, the purpose of this study is to design a culture-sensitive guideline appropriate to the content of the Iranian society, in accordance with the biological, cognitive, psychological and social structure of married adolescent girls in order to empower them in reproductive health. Therefore, it seems that the findings of this study will help policymakers and providers of health services, adolescents and their families to apply the recommendations in the guideline to empowering adolescents in reproductive health.

## Abbreviations

CASP: Critical Appraisal Skill Programs
ICPD: International Conference on Population and Development
MDG: Millennium Development Goals
PICOT: Population Intervention Comparison Outcome Time
RAND: Research and Development
SDG: Sustainable Development Goals
UCLA: University of California at Los Angeles
